# Aptamer-Based Molecular Recognition of Lysergamine, Metergoline and Small Ergot Alkaloids

**DOI:** 10.3390/ijms131217138

**Published:** 2012-12-14

**Authors:** Elsa Rouah-Martin, Jaytry Mehta, Bieke van Dorst, Sarah de Saeger, Peter Dubruel, Bert U. W. Maes, Filip Lemiere, Erik Goormaghtigh, Devin Daems, Wouter Herrebout, François van Hove, Ronny Blust, Johan Robbens

**Affiliations:** 1 Laboratory of Systemic Physiological and Ecotoxicological Research (SPHERE), Department of Biology, University of Antwerp, Groenenborgerlaan 171, Antwerp B-2020, Belgium; E-Mails: jaytry.mehta@ua.ac.be (J.M.); bieke.vandorst@ua.ac.be (B.V.D.); ronny.blust@ua.ac.be (R.B.); 2 Institute of Agricultural and Fisheries Research (ILVO), Ankerstraat 1, Oostende B-8400, Belgium; E-Mail: johan.robbens@ilvo.vlaanderen.be; 3 Faculty of Pharmaceutical Sciences, Ghent University, Harelbekestraat 72, Ghent B-9000, Belgium; E-Mail: sarah.desaeger@ugent.be; 4 Polymer Chemistry and Biomaterials Group, Ghent University, campus Sterre S4, Krijgslaan 281, Ghent B-9000, Belgium; E-Mail: peter.dubruel@ugent.be; 5 Department of Chemistry, University of Antwerp, Groenenborgerlaan 171, Antwerp B-2020, Belgium; E-Mails: bert.maes@ua.ac.be (B.U.W.M.); filip.lemiere@ua.ac.be (F.L.); devin.daems@ua.ac.be (D.D.); wouter.herrebout@ua.ac.be (W.H.); 6 Structural Biology and Bioinformatics Center, Free University of Brussels (ULB), campus de la Plaine, CP206/02, boulevard du Triomphe, Brussels B-1050, Belgium; E-Mail: egoor@ulb.ac.be; 7 Mycothèque de l’Université catholique de Louvain (BCCMTM/MUCL), Mycology, Applied Microbiology, Earth and Life Institute (ELI), Université catholique de Louvain (UCL), Croix du Sud 2 boîte L7.05.06, Louvain-la-Neuve B-1348, Belgium; E-Mail: francois.vanhove@uclouvain.be

**Keywords:** single-stranded nucleic acid, ergot alkaloids, SELEX procedure, surface plasmon resonance

## Abstract

Ergot alkaloids are mycotoxins produced by fungi of the genus *Claviceps*, which infect cereal crops and grasses. The uptake of ergot alkaloid contaminated cereal products can be lethal to humans and animals. For food safety assessment, analytical techniques are currently used to determine the presence of ergot alkaloids in food and feed samples. However, the number of samples which can be analyzed is limited, due to the cost of the equipment and the need for skilled personnel. In order to compensate for the lack of rapid tests for the detection of ergot alkaloids, the aim of this study was to develop a specific recognition element for ergot alkaloids, which could be further applied to produce a colorimetric reaction in the presence of these toxins. As recognition elements, single-stranded DNA ligands were selected by using an iterative selection procedure named SELEX, *i.e.*, Systematic Evolution of Ligands by EXponential enrichment. After several selection cycles, the resulting aptamers were cloned and sequenced. A surface plasmon resonance analysis enabled determination of the dissociation constants of the complexes of aptamers and lysergamine. Dissociation constants in the nanomolar range were obtained with three selected aptamers. One of the selected aptamers, having a dissociation constant of 44 nM, was linked to gold nanoparticles and it was possible to produce a colorimetric reaction in the presence of lysergamine. This system could also be applied to small ergot alkaloids in an ergot contaminated flour sample.

## 1. Introduction

**Figure 1:**
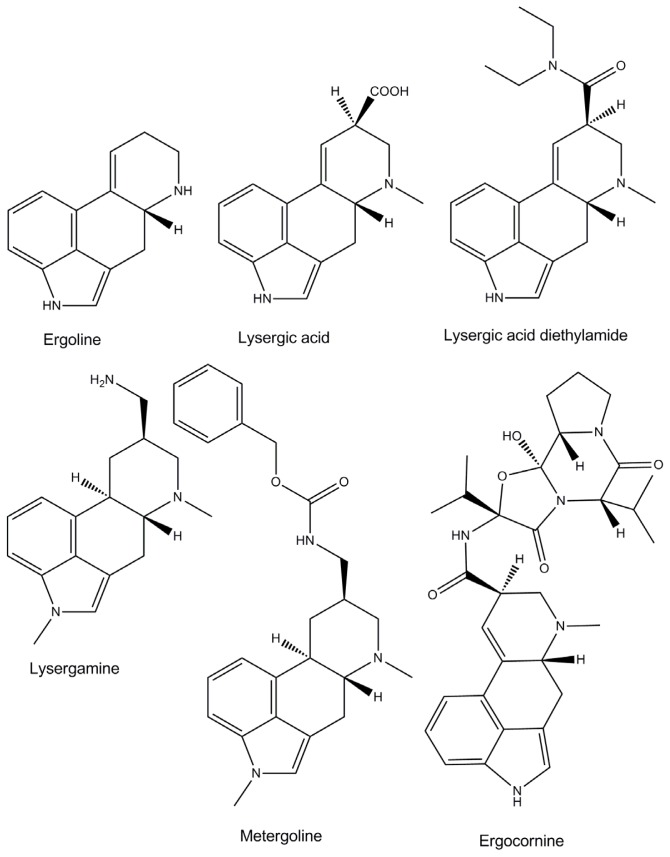
Chemical structures of ergoline and some natural and semi-synthetic ergot alkaloids.

Ergot alkaloids represent a family of more than 40 highly biologically active molecules which can have poisoning effects [1]. Although some ergot alkaloids and semi-synthetic derivatives have interesting medical properties and are used for medical purposes, most of them are toxic and their absorption can be lethal to humans and animals [2–4]. Ergot alkaloids have a common four-membered ring called ergoline, which interacts with adrenergic, serotinergic and dopaminergic receptors [5]. The ergoline skeleton is substituted on position C8, which supports another more or less elaborated chemical group, which is characteristic for each of the different molecules of the family (Figure 1). Ergot contaminated food samples contain a mixture of ergot alkaloids, in varying proportions. The presence of ergot alkaloids in food and feed samples is currently determined by liquid chromatography-mass spectrometry analysis [6]. However, the number of samples which can be analyzed is restricted due to the cost of the equipment and the need for skilled personnel. Therefore, the aim of this study was to offer a simple and reliable detection method for ergot alkaloids in food and environmental samples.

As recognition elements of sensors, aptamers have emerged in the last two decades and have shown an ability to provide reliable sensing tools, and have increasingly gained interest thanks to their advantageous features [7]. Aptamers are artificial ligands, made of nucleic acids or peptides, which can specifically bind to a target molecule or a family of structurally related compounds. The range of possible targets is extremely wide, going from small organic molecules to entire cells or bacteria [8–11]. The elaborate and unique tridimensional structure of aptamers allows them to specifically recognize their targets with a high sensitivity; they can sometimes distinguish between molecules very close in structure to the target molecule. The synthetic production of aptamers then allows the production of billions of different nucleic acid sequences on a large scale and fulfills the research requirements for affinity molecules. Another important aspect of the synthetic production of aptamers is animal-friendliness, in comparison to antibody production, which usually requires extraction from animals. Therefore, aptamers can be considered as innovative recognition tools, offering an ethical and cost-effective alternative to antibodies. Finally, nucleic acid aptamers are stable molecules and can easily be functionalized, allowing their immobilization onto different supports in order to be included as recognition elements in sensors.

Aptamers are obtained by following an iterative selection procedure called SELEX, *i.e.*, Systematic evolution of ligands by EXponential enrichment, which consists of incubation cycles of an aptamer library with the target molecule and recovery of the fraction of the library bound to the target molecule. In this iterative selection procedure, the pool is enriched in binding material, as the unbound fraction of the library used is discarded and the fraction bound to the target molecule is amplified by a polymerase chain reaction (PCR) and then used for other selection cycles [12–15]. The complexes of aptamers and target molecules can be characterized by surface plasmon resonance (SPR), enabling comparison of the binding of the selected aptamers [16]. Further development of the sensors can be carried out by optimizing the binding aptamers.

**Figure 2:**
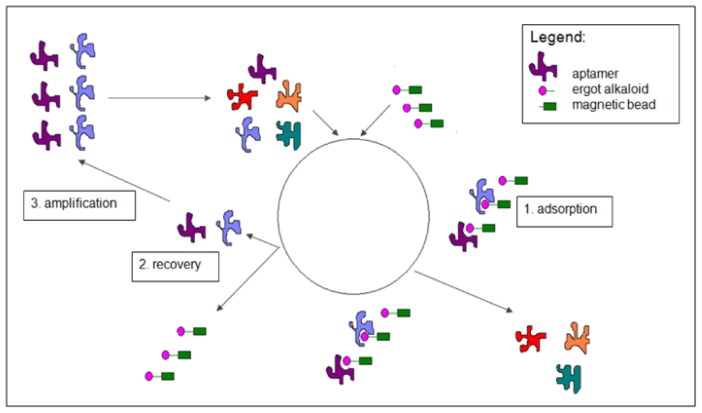
Scheme of SELEX procedure (Systematic Evolution of Ligands by EXponential enrichment) for ergot alkaloids. (**1**) Adsorption. A random 80-base ssDNA library is incubated with the ergot alkaloid coated magnetic beads. By applying a magnetic stand, the fraction of ssDNA which is not bound to the ergot alkaloid coated magnetic beads is discarded. (**2**) Recovery. After washing steps, the fraction of ssDNA bound to the ergot alkaloid coated magnetic beads is eluted from the ergot alkaloid coated magnetic beads. (**3**) Amplification. The selected ssDNA aptamers are amplified by polymerase chain reaction (PCR) in order to be used as input for the following selection cycle.

As ergot alkaloids are a wide group of molecules, it was firstly necessary to decide whether to select an aptamer recognizing specifically one ergot alkaloid, or a more general aptamer able to detect several ergot alkaloids. Considering that the composition of ergot alkaloids can vary enormously from one sample to another, it was decided to work on the chemical part which is common to all the ergot alkaloids, a four-membered ring named ergoline (Figure 1). For this reason, it would have been interesting to work on the natural precursor of ergot alkaloids, lysergic acid, which consists of an ergoline skeleton supporting a carboxylic acid moiety on atom C8 (Figure 1). Unfortunately, lysergic acid, which is also the precursor of the even more potent semi-synthetic lysergic acid diethylamide (LSD), has now been discontinued by most of the chemical firms who had produced it. Moreover, it was not possible to order such a compound in a European country. The focus of this study was therefore directed towards more readily available ergot alkaloids close in structure, metergoline and especially lysergamine (Figure 1). These two ergot alkaloids, metergoline [17] and lysergamine, are mainly composed of an ergoline skeleton, common to all ergot alkaloids, but which is methylated in contrast to the naturally occurring ones. These molecules also contain reactive groups such as a primary amine in the case of lysergamine and a non-fully substituted carbamate moiety in the case of metergoline. For the selection procedure, lysergamine and metergoline were immobilized onto magnetic beads in order to separate the DNA bound to the ergot alkaloids from the rest of the DNA library (Figure 2). Counter-selections were performed with quenched magnetic beads in order to eliminate the non-specifically bound aptamers. The aptamers selected for ergot alkaloids were amplified by PCR and used for the following selection cycles after strand separation of the PCR product. After each selection cycle, the aptamers were inserted into vectors grown in *Escherichia coli* and sequenced after a DNA extraction. The dissociation constants of the complexes of the selected aptamers and lysergamine were determined by SPR. In this analysis, only lysergamine was tested, because its structure is closer to the ergoline ring than metergoline. However, the selection of aptamers for metergoline is reported in this article, because it generated the most sensitive aptamer for the ergoline structure. A colorimetric test was developed by using this aptamer, which was fixed onto gold nanoparticles. The binding of lysergamine or metergoline to the aptamer system with gold nanoparticles resulted in precipitation of the complexes, and a color change occurred. The aptamer-gold nanoparticles system was applied to test a real sample of ergot contaminated flour, for which the same precipitation and color change were observed.

## 2. Results and Discussion

### 2.1. Coating of Magnetic Beads with Lysergamine and Metergoline

**Figure 3:**
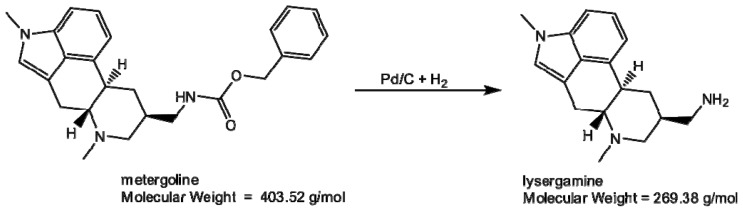
Synthesis of lysergamine by hydrogenolysis of the benzyloxycarbonyl group of metergoline.

**Figure 4:**
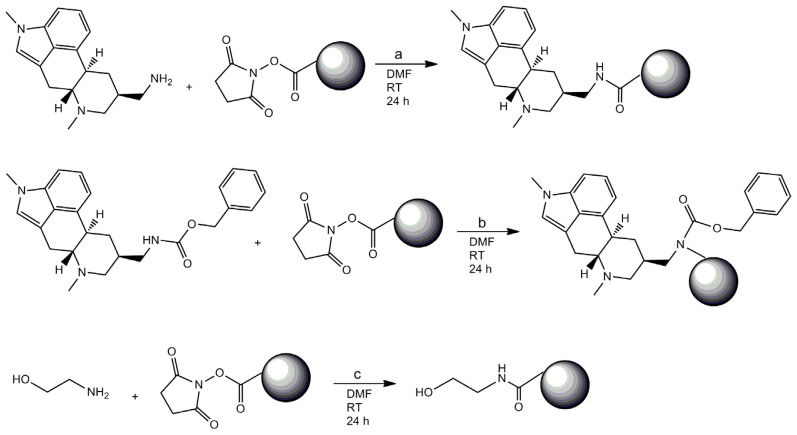
Scheme of the coating of *N*-hydroxysuccinimide-activated (NHS-activated) magnetic beads (**a**) reaction with metergoline (**b**) reaction with lysergamine (**c**) reaction with ethanolamine.

**Figure 5:**
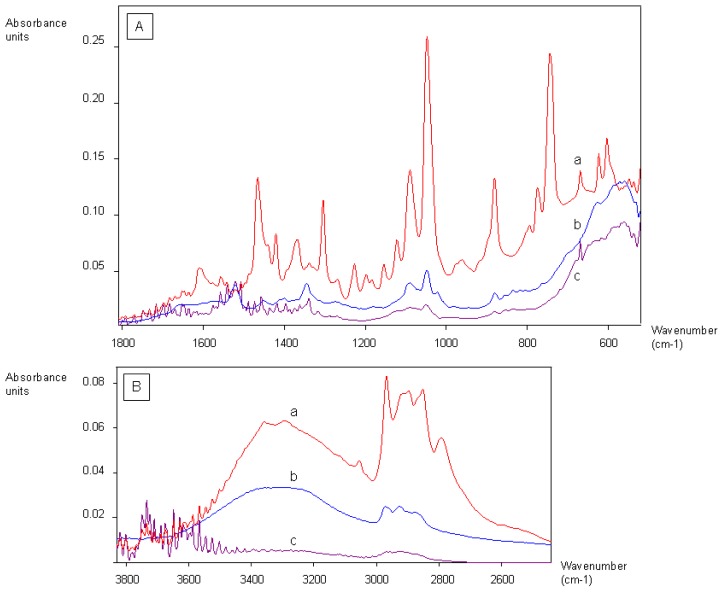
Infrared spectra of the coating of NHS-functionalized magnetic beads with lysergamine. (**A**) Range from 600 cm^−1^ to 1800 cm^−1^ (**B**) Range from 2600 cm^−1^ to 3800 cm^−1^. (**a**) Lysergamine (**b**) Ethanolamine quenched NHS-activated magnetic beads (**c**) Lysergamine coated NHS-activated magnetic beads.

**Figure 6:**
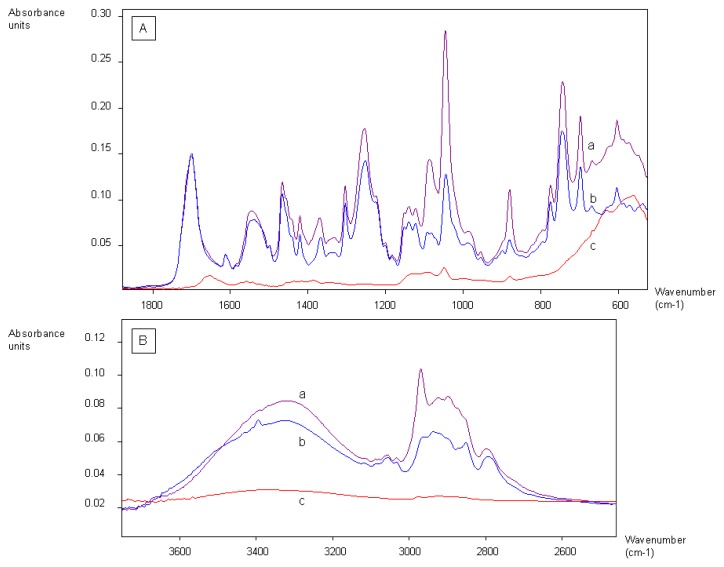
Infrared spectra of the coating of NHS-functionalized magnetic beads with metergoline. (**A**) Range from 600 cm^−1^ to 1800 cm^−1^ (**B**) Range from 2600 cm^−1^ to 3600 cm^−1^. (**a**) Metergoline coated NHS magnetic beads (**b**) Metergoline (**c**) Ethanolamine quenched NHS magnetic beads.

In order to select aptamers, it is usually necessary to immobilize the target molecule onto a support, in order to separate the single-stranded DNA (ssDNA) bound to the target molecule from the rest of the library. Several materials can be used, such as silica columns onto which the target molecule is grafted [18], or magnetic beads [19]. Magnetic beads are convenient supports as they can be used with rather small quantities of material and are easy to handle from a practical point of view. They form a suspension in liquids and they can be incubated in a buffer solution containing the ssDNA library. Then, by applying a magnetic stand, the magnetic beads are gathered onto the wall of the tube and the supernatant containing the unbound fraction of the library can be easily discarded. The weakly bound ssDNA fragments are removed by washing the magnetic beads. The ssDNA bound to the target molecule are eluted from the magnetic beads by applying a chelating elution buffer and heating the solution. In this work, two ergot alkaloids, lysergamine and metergoline, were fixed onto *N*-hydroxysuccinimide-activated (NHS-activated) magnetic beads. Lysergamine had been previously obtained by cleavage of metergoline (Figure 3) in order to obtain a structure closer to that of ergoline, moreover possessing a reactive primary amine moiety [20]. The product resulting from metergoline cleavage was characterized by mass spectrometry (Figure S1). The protonated molecule at *m/z* 270.24 (*m/z* calculated 270.19) was observed in the mass spectrum, confirming that lysergamine was obtained by hydrogenolysis of the benzyloxycarbonyl group of metergoline. The NHS ester groups of the magnetic beads were used to react either with the primary amine of lysergamine or the carbamate moiety of metergoline (Figure 4). A solution of ethanolamine was used to quench the unreacted NHS esters of the magnetic beads. Ethanolamine quenched magnetic beads were also prepared for counter-selections (Figure 4), in order to eliminate the non-specific binding of the aptamers to ethanolamine or the rest of the magnetic beads. The coating of magnetic beads was analyzed by Fourier transform infrared attenuated total reflection (FTIR-ATR) by evaporating concentrated solutions of reagents and products. The advantage of the use of infrared spectroscopy for these types of reaction products is that it enables the analysis of surface coating of the magnetic beads and the determination of the chemical bonds formed or broken. In this way, it is even possible to find out the orientation of the molecules immobilized onto the magnetic beads. Figure 5 shows the infrared analysis of the coupling between the amine group of lysergamine and NHS-beads, which was characterized by the loss of the peaks of the primary amine (ν_max_ cm^−1^ 3298 and 3355) and the formation of an amide bond (ν_max_ cm^−1^ 1679). The stretching of the C–N peak at ν_max_ cm^−1^ 1089 increased in the spectrum of the lysergamine coated beads, due to the formation of the amide bond. The characteristic peaks of lysergamine were found in the spectrum of lysergamine coated beads at ν_max_ cm^−1^: 2968, 2896, 1451, 1422, 1389, 1304, 1049, 880 and 679. The covalent binding of lysergamine occurs on the primary amine which is opposite to the methylated ergoline part. It was then possible to conclude that the methylated ergoline skeleton was situated on the outer part of the magnetic beads, consequently being accessible to the aptamer library in a further step of the selection procedure. Figure 6 shows the infrared spectra obtained from the coating of NHS-functionalized magnetic beads by metergoline, in which the loss of the N-H stretching peak of the carbamate group at ν_max_ cm^−1^ 3400 and an increase of C–N stretching peak at ν_max_ cm^−1^ 1090 due to the new bond formed between the magnetic beads and metergoline, were observed. The characteristic absorption peaks of metergoline were also found in the spectrum of metergoline coated magnetic beads. It was thus possible to determine that the molecules of metergoline were attached to the magnetic beads at the center of the molecules, and that both the ergoline skeleton and the other cyclic part were exposed to the ssDNA library for the selection procedure.

### 2.2. Selection of DNA Aptamers for Lysergamine and Metergoline

**Figure 7:**
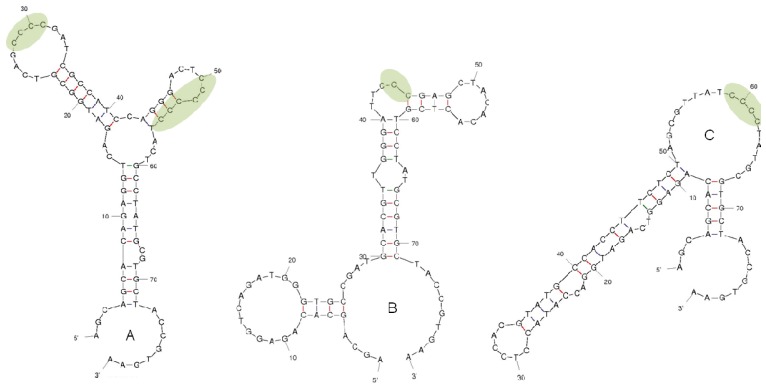
Secondary structures of three aptamers selected for metergoline and lysergamine calculated by Mfold software with the predicted binding sites with common motifs of C bases in green areas. (**A**) Aptamer M3.2 (**B**) Aptamer L5.2 (**C**) Aptamer L5.7.

Ergot alkaloid coated magnetic beads were used for the selection of aptamers from an 80 base ssDNA library, consisting of 40 random nucleotides region flanked by two fixed primer regions of 20 bases. The selections for lysergamine and metergoline coated beads were carried out in parallel. A total of nine selection cycles were performed with metergoline coated beads and seven cycles with lysergamine coated beads. Counter-selections with ethanolamine coated magnetic beads were applied starting from the second selection cycle, in order to remove the non-specifically bound aptamers. After each selection cycle, the recovered ssDNA was amplified and used as input for the following selection cycle. The amount of ssDNA recovered after each selection cycle increased consistently; going from 2% recovery for the first cycle to 87% recovery for the ninth selection cycle for metergoline; and from 3% recovery to 79% recovery after the seventh selection cycle for lysergamine. The resulting aptamers were cloned and sequenced. The dissociation constants were then determined by SPR. The secondary structures of three selected aptamers (Figure 7) were determined by Mfold software [21]. After the last selection cycles with metergoline and lysergamine, it was observed that more than 80% of the selected aptamers had a common motif made of consecutive C bases (from 3 to 6). As a random ssDNA library was used, it was observed that the pools were enriched in C bases after the selection procedure. Therefore, it could be suggested that motifs of consecutive C bases play a role as binding sites in the recognition of lysergamine and metergoline. In the literature, several aptamers were found to be enriched in one of the bases. For instance, G rich aptamers were reported for various target molecules [22–26]. The fact that one of the bases is significantly increased can be linked with the interaction of the aptamer with the target molecule. These regions are usually identified as binding sites or parts of them. For instance, a conformational change of the G-quartet of the thrombin aptamer was observed upon binding [25]. Some other studies on different target molecules have reported slight increments of one of the other nucleic acid bases, such as T or C [27,28].

### 2.3. SPR Analysis of the Complexes of Aptamers and Lysergamine

#### 2.3.1. Coating of SPR Streptavidin Chip with Biotinylated Derivatives of Lysergamine and Ethanolamine

As the focus of the study was to determine the binding between the selected aptamers towards the methylated ergoline part, only lysergamine was used in the SPR analysis. The aptamers obtained from the selections for lysergamine and metergoline were tested. This way, it was possible to discard the aptamers binding to the phenyl part of metergoline. In order to immobilize the target molecule onto a SPR chip, the (strept)avidin/biotin interaction has often been used due to its efficiency and convenience of use for the immobilization of the target molecule. Several coupling reactions can be used to obtain a biotinylated derivative of the target molecule [29]. For this purpose, a biotinylated derivative of lysergamine with a NHS-polyethylene glycol-biotin (NHS-PEG4-biotin) linker was prepared (Figure S2) in order to coat a streptavidin coated SPR chip. An ethanolamine quenched linker was also prepared with a NHS-PEG4-biotin linker and was injected onto a streptavidin SPR chip as a reference (Figure S3). The reaction products, biotinylated linker with lysergamine or ethanolamine, were analyzed using mass spectrometry in positive mode (Figures S4 and S5), in which the protonated reaction product lysergamine-PEG4-biotin was found at *m/z* 743.41 (*m/z* calculated 743.41) and the protonated reaction product ethanolamine-PEG4-biotin was found at *m/z* 535.29 (*m/z* calculated 535.28). These two reaction products were injected into different channels of the SPR chip with the same parameters (concentration, flow, injection time). The different solutions of aptamers were passed through the coated surfaces and the responses were calculated by subtracting the reference measurement from the sample measurement. The SPR responses obtained for the coating with biotinylated lysergamine was in the range of 500 resonance units (RU), and in the range of 350 RU for the biotinylated ethanolamine, which is a smaller molecule and therefore gives a lower response.

#### 2.3.2. SPR Measurements

The dissociation constants of the complexes of lysergamine and aptamers were determined by least-squares fitting of the values of the responses given by the SPR analysis with a non-linear regression of the following equation:

(1) $$\left[y=B_{\text{max}}\times x/(K_{\text{d}}+x)\right]$$

with *y*, the degree of saturation; *B*_max_, the number of maximum binding sites; *K*_d_, the dissociation constant and *x*, the concentration of ssDNA aptamer.

**Figure 8:**
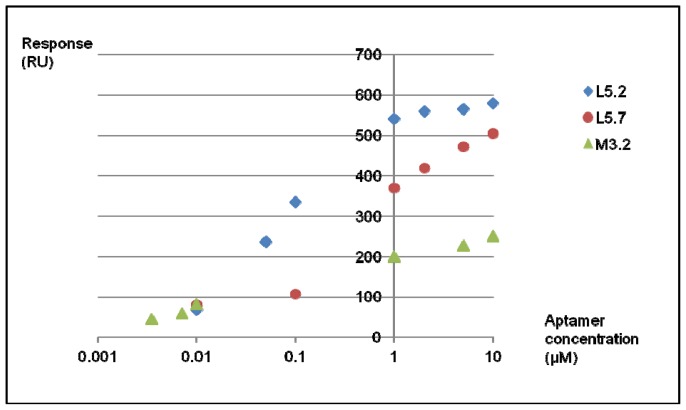
SPR responses of the aptamers binding to lysergamine.

For the SPR analysis, different concentrations of the selected aptamers were injected onto a streptavidin surface coated with lysergamine. The responses obtained for ethanolamine were subtracted from the responses obtained for lysergamine. Triplicates of the measurements were performed with each different aptamer. Other references were tested, such as a non-modified streptavidin surface and a streptavidin surface coated with biotin. However, these two latter references gave lower responses (<5 RU) than the ones obtained for ethanolamine (<10 RU). Therefore, only the ethanolamine coated streptavidin surface was considered for the determination of K_d_s in the binding assay. As shown in Figure 8 and Table 1, aptamer M3.2 had a *B*_max_ of 205.2 RU, representing approximately one third of the values obtained for L5.2 and L5.7, respectively 575.8 and 531 RU. The three selected aptamers had dissociation constants (*K*_d_s) ranging from 44 nM^2^ for aptamer M3.2 to 499 nM for aptamers L5.2 and L5.7 (Table 1). These three aptamers represent sensitive ligands for lysergamine, as the dissociation constants (*K*_d_s) obtained for the complexes are to be found in the nanomolar range. These results are in accordance with those reported in the literature, usually ranging from millimolar to picomolar range for the most sensitive aptamers [9,30]. The best fit for the values obtained for aptamer M3.2 corresponded to a model with two binding sites, instead of a model with one binding site for aptamers L5.2 and L5.7. The fact the aptamer M3.2 gave the lowest dissociation constant (K_d_) and is in accordance with the primary structures of the sequences obtained from the selected aptamers, in which two common motifs of consecutive C bases were found in this aptamer, and the same common motif was present only once in the two other aptamers selected. The value of B_max_ obtained for aptamer M3.2, representing one third of the values obtained for the other aptamers and not a half as expected, can be explained by considering that the binding of this aptamer to two molecules of lysergamine can cover the area of three molecules of lysergamine on the SPR chip.

### 2.4. Colorimetric Reaction with Gold Nanoparticles

**Figure 9:**
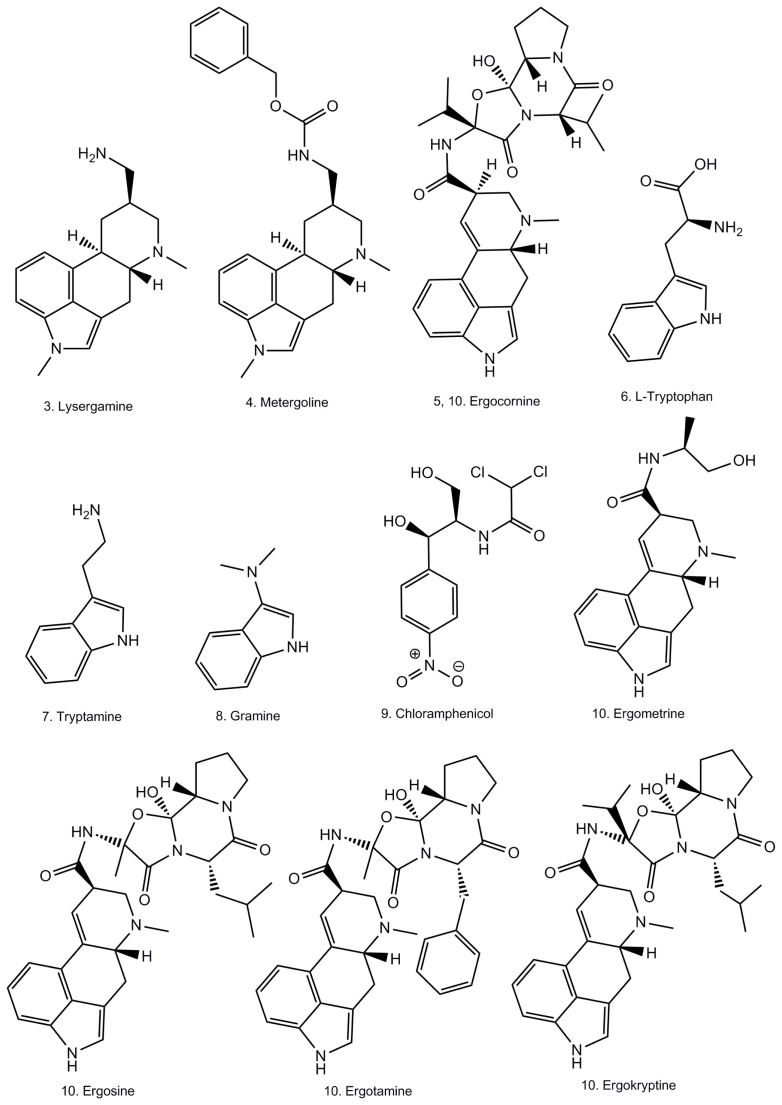
Chemical structures of the compounds tested in the colorimetric reaction assigned with the sample number of the assay.

**Figure 10:**
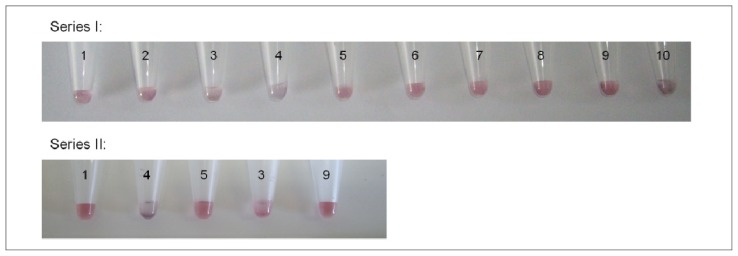
Colorimetric reaction based on gold nanoparticles linked to aptamer M3.2. (1) Reference, no compound added (2) DMF (3) Metergoline (4) Lysergamine (5) Ergocornine (6) L-Tryptophan (7) Tryptamine (8) Gramine (9) Chloramphenicol (10) Ergot contaminated flour sample.

**Figure 11:**
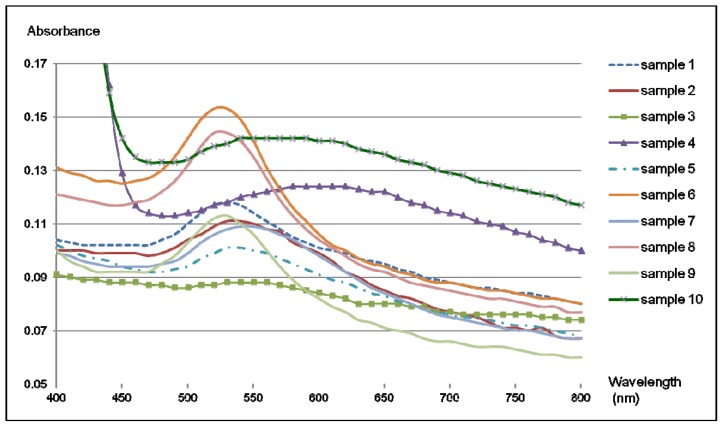
UV-Vis absorbance spectra of samples 1 to 10 from Series I, range from 400 nm to 800 nm.

Aptamers can be linked to gold nanoparticles by following the methodology elaborated by Liu and Lu [31,32] in order to produce a colorimetric reaction in the presence of the target of interest. For this, the selected aptamer is hybridized to two small DNA fragments linked to colloidal gold by a thiol bond. Upon binding to the target molecule, a conformational change in the aptamer can occur. This conformational change can lead to a modification of the chemical environment surrounding the gold nanoparticles, inducing a color change of the solution. Once the modified version of aptamer M3.2 was fixed onto gold nanoparticles, solutions of different ergot alkaloids in dimethylformamide (DMF) were tested: lysergamine, metergoline, ergocornine and an extract of ergot contaminated flour containing several ergot alkaloids such as ergometrine, ergosine, ergotamine, ergocornine, ergokryptine, ergocristine and their epimers in various proportions (Figure 9). As the ergoline skeleton of ergot alkaloids contains a two-membered ring structure named indole, several compounds having an indole group which could be possibly found in food samples, such as L-tryptophan, gramine and tryptamine were also tested (Figure 9). In addition, another molecule differing from ergot alkaloids and containing a phenyl group, chloramphenicol, was also tested (Figure 9). The solutions of the different compounds were prepared in DMF with a concentration of 50 mM and for each sample, 1 μL was taken and placed in the tubes containing the solution of aptamers linked to gold nanoparticles. A rather high concentration of compounds was chosen in order to provide a qualitative assay clear enough to be seen by the naked eye. The colorimetric test was performed with a diluted solution of gold nanoparticles (Series I) and was measured by UV-Vis spectroscopy. A second set of samples was prepared with a more concentrated solution of gold nanoparticles and some of the compounds from Series I. Figure 10 shows the results of the colorimetric assay with aptamer M3.2 linked to gold nanoparticles, in which a precipitate was obtained with lysergamine (sample 3), metergoline (sample 4) and the ergot contaminated flour extract (sample 10); and a color change occurred in the presence of lysergamine and the ergot contaminated flour extract. The color change was much less pronounced in the case of metergoline (sample 3). The other molecules tested: the solvent DMF (sample 2), ergocornine (sample 5), L-tryptophan (sample 6), tryptamine (sample 7), gramine (sample 8), and chloramphenicol (sample 9), did not generate any change. In Figure 11, the UV-Vis spectra are presented of the different solutions of samples 1 to 10. In this analysis, it was observed that the solution of gold nanoparticles linked to aptamer M3.2 (sample 1) had a maximum absorption at 525 nm. The same absorption profile was also obtained for samples 2, 5, 6, 7, 8 and 9; containing respectively: the solvent DMF, ergocornine, L-tryptophan, tryptamine, gramine and chloramphenicol. In the case of lysergamine (sample 4) and the extract of a contaminated sample of flour (sample 10), the maximum absorption was shifted to 600 nm. In the case of metergoline (sample 3), a fading of the absorption peak between 530 nm and 600 nm was observed. From this colorimetric assay, it was possible to conclude that aptamer M3.2 could generate a specific binding reaction in the presence of ergot alkaloids and that it was able to distinguish between the different ergot alkaloids tested. First, in the case of lysergamine, it was suggested that two molecules of lysergamine could saturate the two binding sites of aptamers M3.2, resulting in the formation of a purple precipitate and a shift of the UV-Vis absorption spectrum. In the case of metergoline, which is more voluminous than lysergamine, it was concluded that only one binding site of aptamer M3.2 was occupied, resulting in the formation of a precipitate with a much reduced color change. The difference in behavior towards lysergamine and metergoline is highlighted in Series II (Figure 10), in which a more concentrated solution of aptamers linked to gold nanoparticles was used for the colorimetric assay, enabling the evaluation with the naked eye of the difference in color of the precipitates obtained with these two different molecules. As aptamer M3.2 was originally selected for metergoline, and not lysergamine, it was supposed that one of the two binding sites of aptamer M3.2 was disabled by the linkage to gold nanoparticles in the colorimetric assay and that only smaller molecules than metergoline could fit the second binding site. The sample of ergot contaminated flour extract (sample 10) gave a strong color change and a shift in the absorption spectrum from 525 nm to 600 nm as well as showing lysergamine, even though this sample did not contain any molecules of lysergamine, but a mixture of different natural ergot alkaloids (Table 2). As the sample of ergocornine (sample 5), did not lead to any change, it is supposed that the aptamer did not interact with such a compound in the real ergot sample, nor the other voluminous ergot alkaloids such as ergosine, ergotamine or ergokryptine (Figure 9). It was then concluded that the aptamer M3.2 could interact with small natural ergot alkaloids such as ergometrine (Figure 9), having a structure and size close to those of lysergamine. The interfering compounds having an indole group; such as gramine, L-tryptophan or gramine; did not generate any change. It was then possible to deduce that aptamer M3.2 was specific to the ergoline structure, with or without a methylation on position N1. Regarding specificity, the team of Rankin *et al.*[33] showed that aptamers could reach a very high level of specificity and reported an aptamer distinguishing molecules only differing by a methyl group. In our study, the methylation of the ergoline skeleton was not the discriminating factor, but rather the size of the ergot alkaloid tested. The specificity could be expressed as being restricted to molecules possessing an ergoline ring and smaller in size than metergoline.

## 3. Experimental Section

### 3.1. Coating of Magnetic Beads with Lysergamine and Metergoline

#### 3.1.1. Production of Lysergamine and Preparation of Lysergamine Coated Beads

In order to obtain a more reactive ergot alkaloid, metergoline was transformed into lysergamine (Figure 3) by hydrogenolysis in a Parr apparatus [20]. In a flask, 50 mg of 10% Pd/C (Sigma Aldrich, Bornem, Belgium) were suspended in 15 mL of methanol and 250 mg of metergoline were added after dissolution in 2 mL of methanol. While stirring, the mixture was sparged with a slow stream of hydrogen gas (2.06 Bar) for 16 h at room temperature (RT). The reaction mixture was filtered through celite and the solvent was evaporated under reduced pressure by using a rotary evaporator. As an oil was obtained, ethyl acetate was added to the product and subsequently evaporated, resulting in a white to light yellow powder of lysergamine. The product resulting from metergoline cleavage was characterized using mass spectrometry in positive mode. The sample was prepared by diluting 2 μL of the reaction mixture in 198 μL of acetonitrile. For the analysis, 5 μL of this solution were injected in a 0.2 mL/min flow of acetonitrile with 0.1% formic acid on an Acquity UPLC system (Waters, Elstree, UK). The mass spectra were recorded using electrospray ionization in positive ion mode using a Xevo TQ MS system (Waters, Elstree, UK).

#### 3.1.2. Preparation of Lysergamine Coated Magnetic Beads

A suspension of NHS-activated magnetic beads (Invitrogen, Merelbeke, Belgium) was prepared by washing a quantity of 2 × 10^9^ magnetic beads with 0.3 mL of dry DMF and placing them in 0.5 mL of dry DMF. Then, 5 mg of lysergamine were added, after dissolution in 0.4 mL of dry DMF. The mixture was stirred for 24 h at RT. By applying a magnet, the supernatant was removed and a quench solution of 2 μL of ethanolamine in 0.6 mL of dry DMF was added to the magnetic beads and the mixture was stirred for 5 h at RT. After applying a magnet, the supernatant was discarded; the magnetic beads were then washed 5 times with 0.3 mL of DMF and finally placed in 0.4 mL of DMF.

#### 3.1.3. Preparation of Metergoline Coated Magnetic Beads

In order to coat magnetic beads with metergoline, 5 mg (1.24 × 10^−5^ mol) of metergoline (Sigma Aldrich, Bornem, Belgium) were dissolved in 0.4 mL of dry DMF and placed in a suspension of 2 × 10^9^ NHS-activated magnetic beads (Invitrogen, Merelbeke, Belgium), previously washed with dry DMF and placed in 0.5 mL of dry DMF. The mixture was stirred for 24 h at RT. After applying a magnet, the supernatant containing the excess of metergoline was removed. A solution of 2 μL of ethanolamine in 0.6 mL of DMF was added to the magnetic beads in order to quench the unreacted NHS groups and the mixture was stirred for 4 h at RT. After applying a magnet, the coated magnetic beads were washed 5 times with DMF before being placed in 0.4 mL of DMF.

#### 3.1.4. Preparation of Ethanolamine Coated Beads

Quenched magnetic beads were prepared by adding a solution of 5 μL ethanolamine in 0.6 mL of dry DMF to a suspension of 2 × 10^9^ NHS-functionalized magnetic beads in 0.3 mL of dry DMF. The mixture was stirred for 4 h at RT. By applying a magnet, the supernatant was discarded and the ethanolamine coated magnetic beads were washed 5 times with 0.3 mL of DMF, and were finally placed in 0.4 mL of DMF.

All the coating reactions were characterized by infrared spectroscopy with an FTIR-ATR spectrometer (Vector 22 Brücker, Zürich, Switzerland). For each sample, 80 μL of the coated magnetic bead solutions were taken and after applying a magnetic stand, the solvent was discarded. The coated magnetic beads were washed with ethanol and placed in 10 μL of ethanol. The concentrated solutions of coated magnetic beads in ethanol were evaporated onto the infrared chip for the analysis.

### 3.2. Selection of DNA Aptamers for Lysergamine and Metergoline

#### 3.2.1. Selection Procedure

The first selection cycle was performed directly with the magnetic beads coated with lysergamine or metergoline. All the other selection cycles were performed after a counter-selection with ethanolamine coated magnetic beads. A counter-selection was not performed for the first selection cycle as the amount of ssDNA recovered, which is in the range of 2% of the amount of library used, would be too low to amplify. However, starting from the second selection cycle, the counter-selection step was really crucial to remove the non-specifically binding aptamers. For the first selection cycle, metergoline or lysergamine coated beads were incubated with the ssDNA library. For this, 15 μg of an 80-base DNA library having the following structure 5′-AGCAGCACAGAGGTCAGATG-N40-CCTATGCGTGCTACCGTGAA-3′ (Eurogentec, Seraing, Belgium) was placed in 0.5 mL of binding buffer (100 mM NaCl; 20 mM Tris-HCl pH 7.6; 5 mM KCl; 2 mM MgCl_2_, 6H_2_O; 1 mM CaCl_2_, 2H_2_O) with an amount of 1 × 10^8^ metergoline or lysergamine coated magnetic beads, re-suspended in 0.3 mL of binding buffer. The mixture was gently stirred for 30 min at RT. For the following selection cycles, the new pool of ssDNA was first placed in a suspension of 1 × 10^8^ ethanolamine coated magnetic beads in binding buffer. By applying a magnet, the supernatant containing the ssDNA fraction not bound to the ethanolamine beads was taken and placed in a suspension of 0.1 mL of 1 × 10^8^ ergot alkaloids coated magnetic beads in binding buffer. The mixture was gently stirred for 40 min at RT.

#### 3.2.2. Elution of ssDNA

After applying a magnet, the supernatant was discarded and the beads were washed twice with the binding buffer. The ssDNA fraction bound to the ergot alkaloid coated magnetic beads was eluted with 3 × 0.2 mL of elution buffer (3.5 M urea, 40 mM Tris-HCl, 10 mM EDTA, 0.02% Tween 20) by stirring gently (300 rpm) for 10 min at 80 °C (Eppendorf Thermomixer, Rotselaar, Belgium).

#### 3.2.3. Precipitation of ssDNA

The eluted ssDNA was precipitated by adding 60 μL of 3 M sodium acetate (10% volume of elution buffer) and 1 mL of ice-cold ethanol. This solution was placed at −80 °C for 1 h to ensure complete precipitation. The solution was centrifuged at 13,000 rpm at 4 °C for 20 min in order to concentrate the DNA. The supernatant was discarded leaving approximately 50 μL of solution in the tube. Then, 1 mL of 70% ice-cold ethanol in water was added and the solution was centrifuged at 13,000 rpm at 4 °C for 25 min. Again, the supernatant was discarded leaving 50 μL of solution in the tube and this latter step was repeated twice. The ethanol solution was evaporated in a speed vacuum device at 30 °C for 10 min. The resulting DNA pellet was re-suspended in 15 μL of water (18.2 megohms/cm). The amount of ssDNA obtained from the selection was analyzed with a UV-Vis spectrophotometer (Nanodrop, Erembodegem, Belgium) in order to determine the recovery rate.

#### 3.2.4. Amplification of the selected ssDNA aptamers by PCR

The ssDNA recovered from the selection procedure was amplified by PCR by using a thermostable *Taq* polymerase, PCR buffer, 10 mM dNTP solution and 25 mM MgCl_2_ solution (Fermentas, St Leon-Rot, Germany). The set of primers used for the PCR: 5′-AGCAGCACAGAGGTCAGATG-3′ (forward primer) and 5′-TTCACGGTAGCACGCATAGG-3′ (reverse primer), was provided by Eurogentec (Seraing, Belgium). In each vial, 6 μL of the ssDNA solution were placed in 20 μL water (18.2 megohms/cm). Then, the following solutions were added to the DNA template: 5 μL of PCR buffer, 3.5 μL of 10 mM dNTP, 11 μL of 25 mM MgCl_2_, 0.5 μL of 100 μM solution of forward primer, 0.5 μL of 100 μM solution of reverse primer and 0.8 μL of Taq polymerase (5 U/μL). The mixture was placed in a thermocycler (Mastercycler personal Eppendorf VWR, Leuven, Belgium) with the following temperature profile: heating at 94 °C over 6 min for the initial denaturation step, followed by 18 cycles of 94 °C for 30 s, 55 °C for 30 s, 72 °C for 20 s, followed by a final elongation step of 72 °C over 1 min 30 s. The Taq polymerase was added to the PCR reaction mixture after 4 min of the initial denaturation step. A gel-electrophoresis of the PCR product was performed after each PCR. A 2% agarose gel was prepared in 1× Tris-acetate-EDTA (TAE) buffer with 10 μL of ethidium bromide for 100 mL of buffer. In each well of the agarose gel, 20 μL of PCR solution were placed after staining with a loading dye. A 50 base pair DNA ladder was used to check the length of the PCR product.

#### 3.2.5. Preparation of ssDNA from Biotinylated PCR Product

A part of the double-stranded DNA obtained from the PCR was amplified with a reverse biotinylated primer (Eurogentec, Seraing, Belgium) in order to separate the strands of the PCR product by using streptavidin magnetic beads (Dynabeads M-280 Streptavidin, Invitrogen, Merelbeke, Belgium), with a ratio of 0.1 mg of magnetic beads for 1 μg of dsDNA. The streptavidin magnetic beads were washed with 1× binding and washing (B & W) buffer (5 mM tris-HCl pH 7.5, 0.5 mM EDTA, 1 M NaCl) and resuspended in 2× B & W buffer to a concentration of 5 μg/μL. An equal amount of water solution of dsDNA was added to the solution of the streptavidin magnetic beads and the mixture was gently stirred for 25 min at RT. By applying a magnet, the magnetic beads were gathered onto the tube wall and the supernatant was discarded. The beads were washed twice with 1× B & W buffer and re-suspended in 0.15 M NaOH for 5 min at 37 °C in order to release the non-biotinylated DNA strand. After applying a magnet, the solution was placed in a clean vial and immediately neutralized with an equal amount of 0.15 M acetic acid. The DNA was precipitated by adding 10% volume of 3 M sodium acetate and ice-cold ethanol to give a ratio of 70% in the final solution. This solution was incubated at −80 °C for one hour and then centrifuged at 13,000 rpm at 4 °C for 20 min. The solution was discarded; leaving approximately 50 μL at the bottom of the tube and 70% ethanol was added to the DNA pellet. The solution was centrifuged again with the same conditions and the step was repeated. The supernatant was discarded, leaving 50 μL of solution, which was evaporated in a speed vacuum device (Eppendorf Concentrator 5301, VWR, Leuven, Belgium). The ssDNA was re-suspended in water and the concentration was determined by UV-Vis spectroscopy (Nanodrop, Thermo scientific, Erembodegem, Belgium).

#### 3.2.6. Cloning and Sequencing of Aptamers

After each selection cycle, a part of the ssDNA selected was amplified by PCR with non-modified primers and cloned into the vector pCR2.1-TOPO (TOPO TA cloning kit, Invitrogen, Merelbeke, Belgium) and transformed in *Escherichia coli* TOP 10 competent cells. The colonies containing the aptamer sequences were picked and the plasmid DNA was extracted by using a plasmid extraction kit (QIAprep Spin Miniprep kit, Qiagen, Venlo, Netherlands). After DNA purification, the plasmid DNA was sequenced (VIB Genetic Service Facility, Antwerp, Belgium). The secondary structures of the aptamers were determined using Mfold software developed by Zucker [21] in order to calculate the lowest energy levels of the secondary structures formed by the aptamers.

### 3.3. SPR Analysis of the Complexes of Aptamers and Lysergamine

#### 3.3.1. Preparation of Biotinylated Lysergamine Linker

In order to prepare the biotinylated derivative of lysergamine to be used with streptavidin SPR chip, 6 mg (2.22 × 10^−5^ mol) of lysergamine were dissolved in 0.25 mL of dry DMF and a solution of 2 mg (3.4 × 10^−6^ mol) of NHS-PEG4-biotin linker (EZ-Link, Thermo Scientific, Belgium) in 0.25 mL of dry DMF was added. The mixture was stirred for 24 h at RT.

#### 3.3.2. Preparation of Biotinylated Ethanolamine Linker

As a reference material, a NHS-PEG4-linker quenched with ethanolamine was prepared by dissolving 3 mg (4.97 × 10^−5^ mol) of ethanolamine (Sigma Aldrich, Belgium) in 0.25 μL of dry DMF and adding 2 mg (3.4 × 10^−6^ mol) of NHS-PEG4-biotin linker dissolved in 0.25 mL of dry DMF. The reaction mixture was stirred for 5 h at RT.

The products obtained from the reaction between the biotinylated linker and lysergamine or ethanolamine were characterized using mass spectrometry by placing 2 μL of the reaction mixtures in 198 μL of acetonitrile. For each sample, 5 μL were injected into a 0.2 mL/min flow of acetonitrile with 0.1% formic acid on an Acquity UPLC system (Waters, Elstree, UK). The mass spectra were recorded using electrospray ionization in positive ion mode on a Xevo TQ MS system (Waters, Elstree, UK).

#### 3.3.3. Coating SPR Chip with Biotinylated Linkers

As streptavidin supports restrict the use of organic solvents, 75 μL from each of the reaction mixtures were diluted in 300 μL of HBS-EP buffer (10 mM HEPES pH 7.4, 150 mM NaCl, 3.4 mM ethylenediaminetetraacetic acid (EDTA), 0.005% surfactant P20), in order to obtain a ratio of 25% of DMF in the final solution, which was then injected into the SPR chip (Biacore GE Healthcare, Diegem, Belgium). In the SPR device (Biacore, GE Healthcare, Diegem, Belgium), a streptavidin chip was used and 120 μL of the two different coating solutions were injected with a flow of 5 μL/min.

After the coating reaction, the HBS-EP buffer was replaced by a continuous flow of binding buffer and the solutions of the different aptamers in binding buffer were loaded onto the coated SPR chip. The lysergamine coated surfaces were regenerated by applying pulses of 5 M urea.

The analysis of the binding of ssDNA aptamers to the lysergamine coated surface was compared with the binding to the ethanolamine coated surface, which was the most relevant reference for this analysis. However, several other references were tested such as a biotin coated streptavidin surface and a non-modified streptavidin surface.

### 3.4. Colorimetric Reaction with Aptamer Linker to Gold Nanoparticles

The procedure developed by Liu and Lu [31,32] was followed with the aptamer selected for ergot alkaloids. The following labeled DNA sequences (Eurogentec, Belgium) were used in the colorimetric reaction: 5′-ACTCATCTGTGAAGAGAAGCAGCACAGAGGTCAGATGTCCGTCAGCCCCGATCGCCATCCAGGGACTCCCCCCTATGCCTATGCGTGCTACCGTGAA-3′ (modified aptamer M3.2), 5′-TCACAGATGAGT-C3-SH-3′ (3′-SH oligo) and 5′-SH-C6-TGCTGCTTCTCT-3′ (5′-SH oligo). In a microcentrifuge tube were placed 1.6 μL of 1 mM 3′-SH oligo and 7.4 μL of water. In a second microcentrifuge tube, 1.6 μL of 1 mM 5′-SH oligo was placed in 7.4 μL of water. Then, 1 μL of 500 mM acetate buffer (pH 5.2) and 1.5 μL of tris(2-carboxyethylphosphine) TCEP-HCl (Perbio Science, Erembodegem, Belgium) were added to each tube to activate the thiol moiety. After 1 h of activation, 3 mL of 15 nm colloidal gold solution were added to each tube containing TCEP-activated oligos and the mixtures were gently shaken. The tubes were stored in a drawer for 24 h. Then, 30 μL of 500 mM Tris acetate buffer (pH 8.2) were added dropwise to each tube, followed by the addition of 300 μL of 1 M NaCl. The tubes were stored for another day. The functionalized particles were centrifuged at 13,000 rpm for 15 min at RT. The supernatant was discarded and the particles were dispersed in 200 μL of 100 mM NaCl buffer. The two solutions of functionalized gold nanoparticles were mixed together. Then, 10 μL of 10 μM modified aptamer M3.2 were added and the mixtures were incubated at 4 °C overnight. The aggregates were centrifuged at 2000 rpm for 1 min at RT. The supernatant was discarded and the aggregates were dispersed in 500 μL of 300 mM NaCl buffer. For the optimization of salt concentration, 50 μL of the aggregates solution were taken for each analysis. The phase transition of the aptamer-gold nanoparticle aggregates was studied by recording the UV absorption at 260 nm as a function of the temperature with different NaCl concentrations (from 150 mM to 300 mM) in order to find the appropriate NaCl concentration for which the UV-Vis absorption increases sharply at RT. An optimal concentration of 250 mM NaCl was found, as the melting temperature obtained with this parameter was the closest to RT (~23 °C). For the final test, two series of samples with different concentrations of gold nanoparticles solutions were prepared: a concentrated solution (Series II), in which 30 μL of aggregates solution were taken and the NaCl concentration adjusted to 250 mM by adding 100 mM NaCl buffer; and a more diluted series of 40 μL of solution of gold nanoparticles diluted twice (Series I), with a final NaCl concentration of 250 mM. For the specificity test, each compound was prepared in a solution of DMF with a concentration of 50 mM and 1 μL of each of the solutions was added to the test tubes containing the aptamer-gold nanoparticles system. The sample of ergot contaminated flour was obtained by adding 300 mg of contaminated flour sample, provided by the European Food Safety Authority (sample EFSA/82/RE), to 500 μL of DMF and vortexing for 1 min. In this way, the ergot alkaloids were extracted from the flour sample and 1 μL of the solution was used for the test. A preliminary test with DMF only was also performed and did not show any effect on the aptamer-gold nanoparticles system. The UV-Vis spectra of the samples from Series I were measured with a Synergy Mx UV-Vis spectrophotometer (Biotek, Bad Friedrichshall, Germany), and were analyzed using Gen5 software.

## 4. Conclusions

In order to produce a recognition element for ergot alkaloids which can be included in a biosensor, aptamers were selected for two ergot alkaloids, metergoline and lysergamine, by using the iterative selection procedure SELEX. It was possible to isolate, amplify and sequence aptamers binding to metergoline and lysergamine. The complexes of the selected aptamers and lysergamine were characterized by SPR. The selected aptamers have dissociation constants in the nanomolar range, the best one reaching 44 nmol^2^/L^2^ for aptamer M3.2. This aptamer possesses two common motifs of C bases, predicted to be two binding sites. The mathematical analysis confirmed the presence of two binding sites with a lower B_max_ and a lower K_d_ than the other aptamers tested. This aptamer, which has a high sensitivity for lysergamine, is the first one to our knowledge reported for ergot alkaloids. A colorimetric reaction could be achieved with aptamer M3.2 linked to gold nanoparticles and a specific color change was observed in the presence of lysergamine and small ergot alkaloids. This system can be developed further with the construction of a dipstick test using lateral flow diffusion. Other transducing techniques (electrochemical, piezoelectric, optical, *etc.*) can be used with this aptamer as recognition element for the development of different sensing formats. We believe in the potential of this aptamer for applications in the future regarding the detection of small ergot alkaloids.

## Supplementary Information



## Figures and Tables

**Table 1:** Calculations by GraphPad Prism of the dissociations constants (*K*_d_s) of complexes of lysergamine and the selected aptamers.

Fitting Model	Two-site specific binding	One-site specific binding	
Best fit values for	Aptamer M3.2	Aptamer L5.2	Aptamer L5.7
*B*_max_ (RU)	205.2	585.8	531.0
*K*_d_	44 nmol^2^/L^2^	73 nmol/L	499 nmol/L
*R*^2^	0.997	0.993	0.991
Number of points analyzed	6	7	6
Degree of freedom	2	5	4

**Table 2:** Ergot composition of ergot contaminated flour sample (sample 10), determined by LC-MS [34].

Ergot alkaloid	Concentration (μg/kg)	Total epimers	% of ergot alkaloid content
Ergometrine	106	132	4.3
Ergometrinine	26		

Ergosine	1567	1567	51
Ergosinine	<LOQ		

Ergotamine	22	24	0.8
Ergotaminine	2		

Ergocornine	65	84	2.7
Ergocorninine	19		

Ergokryptine	1251	1259	41
Ergokryptinine	8		

Ergocristine	<LOQ	3	0.1
Ergocristinine	3		

Total	3068	3068	100
