# Supplementary material for: Aptamer-Based Molecular Recognition of Lysergamine, Metergoline and Small Ergot Alkaloids
Source: Int J Mol Sci. 2012 Dec 14;13(12):17138–59. doi: 10.3390/ijms131217138 (PMC3546743; doi:10.3390/ijms131217138)
Supplement: Supplementary file 1 [file ijms-13-17138-s001.pdf]

Supplementary Information

Figure S1. Mass spectrum in positive mode of the reaction product obtained from the cleavage of metergoline into lysergamine.

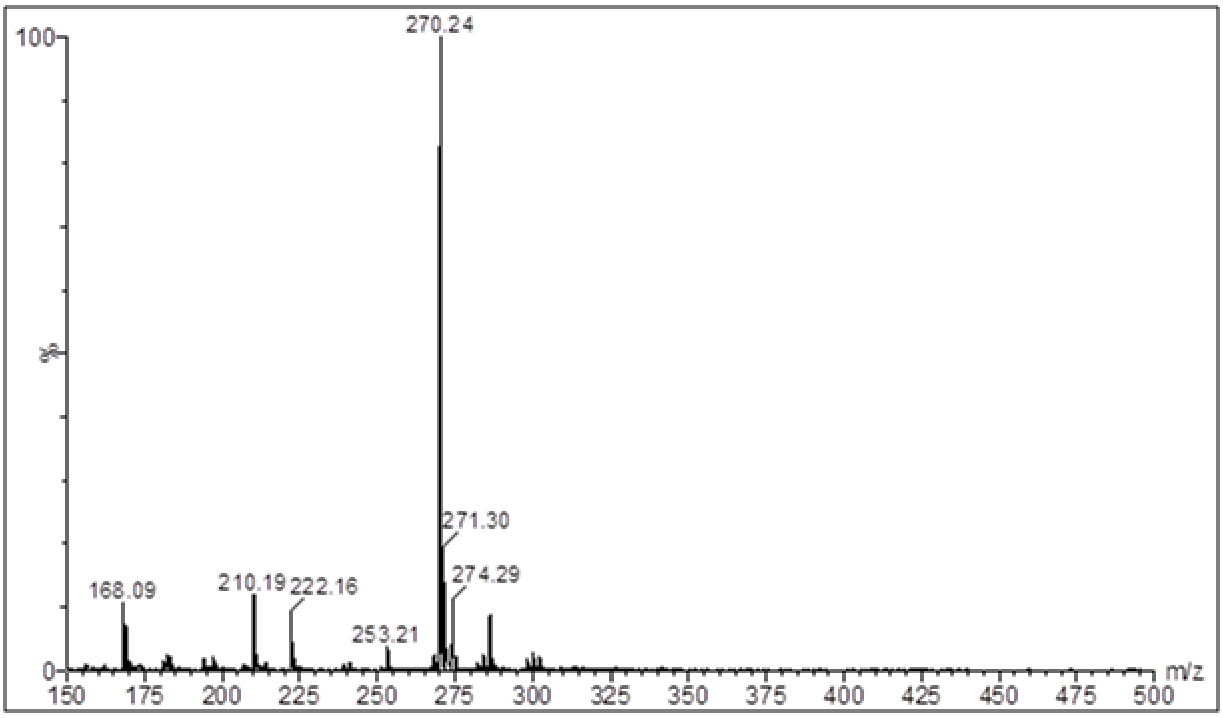

Figure S2. Scheme of the reaction between lysergamine and NHS-PEG4-biotin linker.

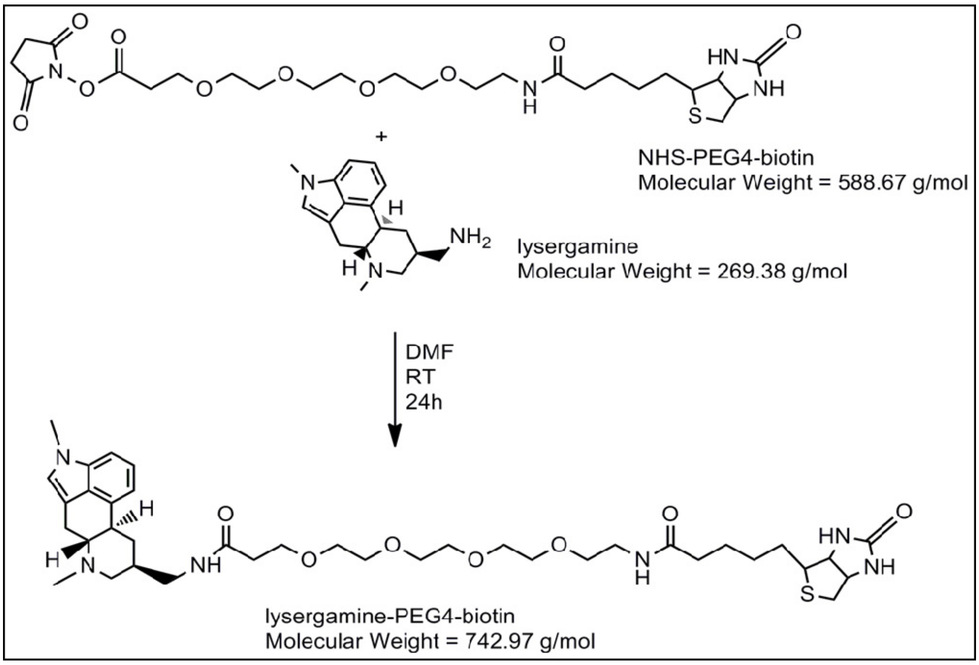

Figure S3. Scheme of the reaction between ethanolamine and NHS-PEG4-biotin linker.

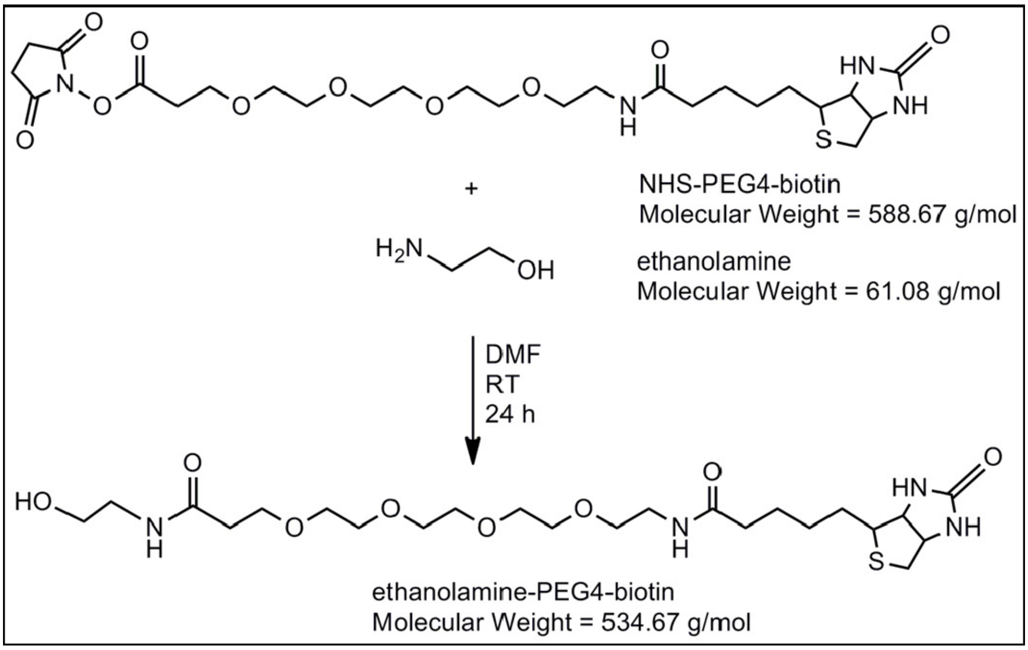

Figure S4. Mass spectrum in positive mode of the product obtained from the reaction between lysergamine and NHS-PEG4-biotin linker.

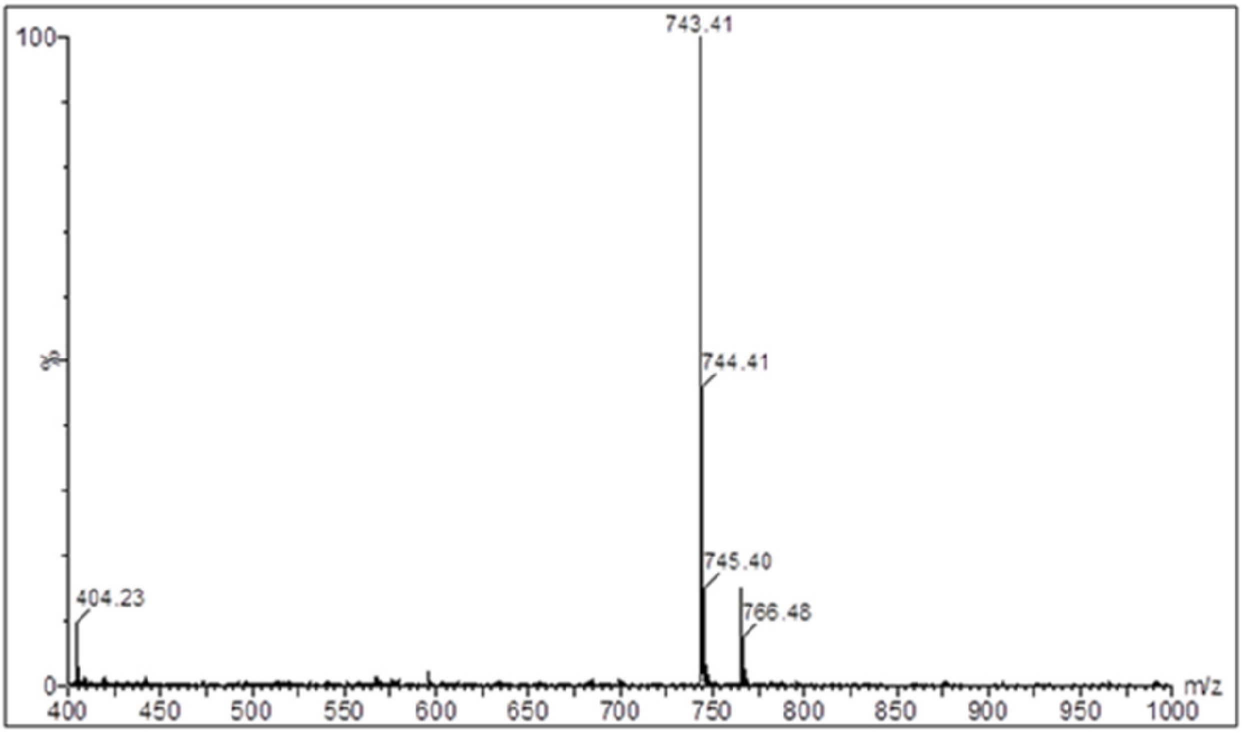

Figure S5. Mass spectrum in positive mode of the product obtained from the reaction between ethanolamine and NHS-PEG4-biotin linker.

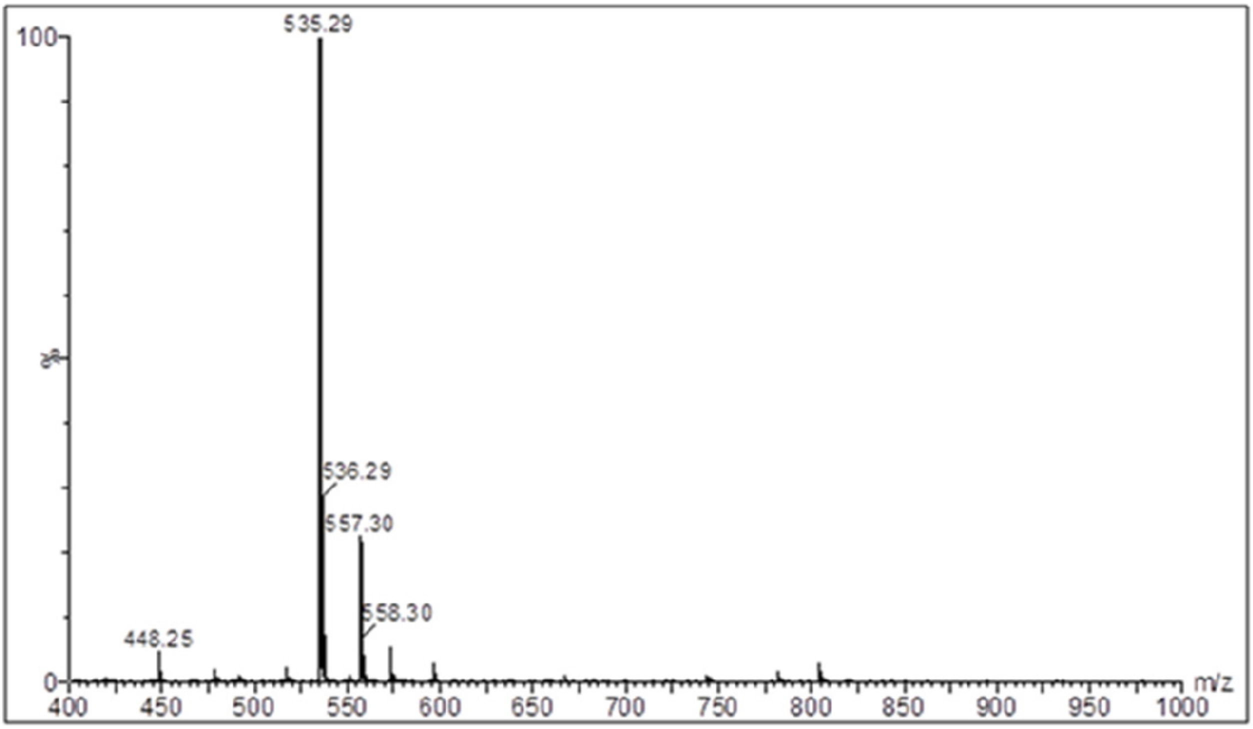

© 2012 by the authors; licensee MDPI, Basel, Switzerland. This article is an open access article distributed under the terms and conditions of the Creative Commons Attribution license (http://creativecommons.org/licenses/by/3.0/).
